# Relation-aware dynamic knowledge subgraph enhanced large language models for medical multiple-choice question answering

**DOI:** 10.1371/journal.pone.0349628

**Published:** 2026-10-16

**Authors:** Yu Ma, Tianning Di, Jiangpeng Xu, Yue Wu

**Affiliations:** 1 Gansu Police College, Lanzhou, China; 2 Police Embodied Intelligence and Unmanned Systems Innovation Center, Lanzhou, China; 3 The Second Hospital of Lanzhou University, Lanzhou, China; 4 Gansu Provincial Public Security Department, Lanzhou, China; Alma Mater Studiorum Universita di Bologna: Universita degli Studi di Bologna, ITALY

## Abstract

Medical multiple-choice question answering requires models to simultaneously understand question semantics, candidate-answer differences, and structured relations among medical entities in complex clinical contexts. To address the limitations that existing knowledge-enhanced methods often adopt question-level unified modeling and thus struggle to characterize local relational differences among different candidate options, while conventional low-rank adaptation lacks relation-pattern adaptivity, this paper proposes a knowledge-enhanced large language model framework that combines option-level knowledge subgraph representation fusion with relation-aware dynamic LoRA adaptation. The proposed method first constructs a local medical knowledge subgraph for the question stem and each candidate answer separately, and then injects structured knowledge into candidate representations through a graph encoder and gated fusion mechanism. Subsequently, multiple learned LoRA expert branches are dynamically combined through candidate-conditioned routing based on the relational information encoded in the enhanced candidate representations, enabling the model to obtain more suitable parameter update paths. The experimental results are reported as the mean and standard deviation over three random-seed runs. On the MedQA dataset, the proposed method achieves Accuracy scores of 62.90%±0.82% and 62.84%±1.04%, together with F1-Score values of 62.97%±1.07% and 62.79%±0.78%, using Llama3 8B and Qwen 7B, respectively. On the MedMCQA dataset, the corresponding Accuracy scores are 61.04%±0.30% and 58.59%±0.65%, while the F1-Score values reach 61.21%±0.54% and 57.77%±0.57%, respectively. Experimental results show that the proposed method can effectively improve knowledge understanding, candidate discrimination, and reasoning stability in medical multiple-choice question answering.

## 1 Introduction

Medical question answering is an important research direction in medical artificial intelligence, aiming to accurately understand question intent, identify key medical concepts, and infer the option that best conforms to medical knowledge and clinical logic from multiple candidate answers in complex medical contexts. With the strong capabilities of large language models in natural language understanding, knowledge memorization, and reasoning generation, their application value in medical question answering, clinical decision support, and medical education has continued to increase [1]. However, medical questions usually involve complex relations among symptoms, diseases, drugs, examinations, pathological mechanisms, and treatment strategies, and relying only on the internal parametric memory of language models is insufficient to fully ensure the stability of the reasoning process and the consistency of medical knowledge [2]. Therefore, how to effectively integrate structured medical knowledge with the semantic representation capability of large language models has become an important research issue for improving the reliability and interpretability of medical question answering.

Although existing medical large language model methods can improve task adaptation through parameter-efficient fine-tuning or external knowledge enhancement, several limitations remain [3]. Previous LM–GNN approaches, including QA-GNN [4], GreaseLM [5], and DRAGON [6], have demonstrated the effectiveness of constructing local knowledge subgraphs associated with question–answer pairs and integrating graph representations with language-model representations for knowledge-intensive question answering. These studies establish an important foundation for fine-grained graph–text reasoning. However, the structured relational information obtained from these local subgraphs is mainly used to enhance representation learning and answer reasoning, while its potential role in dynamically controlling parameter-efficient adaptation paths remains insufficiently explored. Second, distractor options in medical multiple-choice question answering often share certain semantic similarities with the question stem, and relying solely on textual semantic matching can easily blur the discriminative boundary among candidate options [7]. Third, conventional low-rank adaptation methods usually adopt a unified parameter update direction, making it difficult to adapt to heterogeneous relation patterns corresponding to different medical reasoning types, such as diagnosis, medication, examination, and complication discrimination. Therefore, an important remaining challenge is how to couple candidate-specific structured medical relations with dynamic parameter adaptation, so that the relational evidence extracted from knowledge subgraphs can influence not only semantic representations but also the parameter update paths used for different reasoning patterns. These issues limit the model’s ability to capture fine-grained medical relations and weaken its reasoning credibility in complex medical question answering scenarios [8,9].

To address the above issues, this paper proposes a knowledge-enhanced large language model framework for medical multiple-choice question answering, which achieves joint optimization from structured knowledge representation to parameter update paths through the collaborative modeling of option-level knowledge subgraph representation fusion and relation-aware dynamic low-rank adaptation. Specifically, the model first performs medical entity recognition and concept linking on the question stem and each candidate answer, and constructs local medical knowledge subgraphs around different candidate answers, so that each option can preserve its own independent medical relational evidence. Then, the model uses a graph encoder to extract structured knowledge representations related to candidate options, and aligns and fuses them with the textual semantic representations generated by the large language model through a gated fusion mechanism, thereby forming candidate answer representations that contain both linguistic semantics and medical relational information. On this basis, the model further dynamically combines multiple relation-specific low-rank adaptation branches according to the dominant medical relation patterns triggered by candidate options, enabling different medical reasoning scenarios to obtain more suitable parameter update paths.

The main contributions of this paper can be summarized as follows:

- Building on prior option-level knowledge subgraph modeling paradigms, we develop a candidate-specific medical knowledge representation mechanism that explicitly captures fine-grained relational differences between question-stem entities and candidate entities. More importantly, the resulting structured relation representations are further used as conditional evidence for subsequent relation-aware parameter adaptation, extending their role beyond representation enhancement alone.
- A relation-aware dynamic low-rank adaptation mechanism is designed to adaptively combine multiple low-rank adaptation branches according to the medical relation patterns corresponding to candidate answers, thereby alleviating the representation conflicts caused by sharing a unified parameter update direction across heterogeneous medical reasoning types.
- A medical question answering modeling framework that coordinates structured knowledge representation and dynamic parameter adaptation is constructed, enabling medical knowledge subgraphs not only to enhance the semantic representations of candidate answers, but also to provide relational evidence for parameter adaptation path selection, thereby improving the model’s medical knowledge understanding ability, candidate option discrimination ability, and reasoning interpretability in medical multiple-choice question answering.

## 2 Related Work

### 2.1 Knowledge graph enhanced large language models for medical question answering

Knowledge graph-enhanced large language model research for medical question answering has gradually evolved from early knowledge retrieval and matching paradigms toward structured knowledge injection paradigms oriented to generative reasoning. Early studies focused more on constructing domain-specific knowledge question answering systems or knowledge matching frameworks. For example, Liu et al. [10] developed an integrated question answering and information retrieval system based on a traditional Chinese medicine knowledge graph, demonstrating the application potential of structured medical knowledge in vertical-domain question answering. Chen et al. [11] improved semantic alignment in medical question answering through a knowledge abstraction matching mechanism, providing a foundational idea for subsequent knowledge-enhanced models. With the rapid development of large language models, the research focus has further shifted toward how to effectively couple external medical knowledge with the deep representations of pre-trained language models. Yang et al. [12] improved medical question answering performance by combining knowledge graphs with ranking techniques, showing that external structured knowledge can alleviate the limitations of pure language models in medical fact discrimination. The medical knowledge-augmented large language model proposed by Cabello et al. [13] further demonstrated that introducing external knowledge into the question answering process helps improve medical relevance and response reliability. Zafar et al. [14] emphasized, from the perspective of medical answer accuracy, the importance of knowledge graphs in reducing factual errors and enhancing answer credibility. Overall, this line of research has evolved from simple knowledge-assisted retrieval into a unified modeling framework for complex medical semantic understanding and reasoning.

Although existing studies have demonstrated that knowledge graphs significantly promote medical question answering, limitations remain in terms of knowledge injection granularity, relational modeling depth, and collaborative alignment with the parameter adaptation of large language models. On the one hand, some methods still mainly rely on question-level knowledge enhancement or external knowledge textualization and concatenation, making it difficult to characterize the local medical relational differences between the question stem and different candidate answers in a fine-grained manner. To address this issue, Zeng et al. [15] enhanced large language models through knowledge subgraphs, providing a valuable direction for fine-grained subgraph modeling. Chen et al. [16] further injected multi-hop medical knowledge into smaller-scale language models, indicating that complex relational chain modeling is also crucial for biomedical question answering. Closely related LM–GNN studies have further demonstrated the effectiveness of constructing candidate-specific or question–answer-pair knowledge subgraphs and integrating them with language representations. QA-GNN [4] constructs a knowledge subgraph associated with each question–answer context and performs joint reasoning over language-model and graph representations. GreaseLM [5] further introduces iterative interaction between language and graph representations and evaluates this graph–text reasoning paradigm on MedQA using medical knowledge graphs. DRAGON [6] extends this direction through bidirectional language–knowledge graph pretraining and demonstrates its applicability to biomedical question answering with medical knowledge resources. On the other hand, recent studies have also begun to explore the integration of knowledge enhancement with more sophisticated reasoning mechanisms. For instance, Zafar et al. [17] combined first-order logical reasoning with knowledge injection to improve the medical reasoning capability of the model. Rezaei et al. [18] highlighted the role of agentic medical knowledge graphs in connecting large language models with dynamically evolving medical knowledge, reflecting that the static knowledge enhancement paradigm is expanding toward a more interactive and time-sensitive direction. Li et al. [19] further extended knowledge graph enhancement to multilingual medical question answering scenarios, showing that this direction has strong cross-lingual adaptation potential. Although these studies have substantially advanced fine-grained graph–text reasoning, candidate-specific knowledge subgraphs are primarily used to enhance representation learning and answer reasoning. Their structured relational evidence is rarely further exploited to dynamically control parameter-efficient adaptation paths according to heterogeneous medical relation patterns. Therefore, how to couple fine-grained knowledge subgraph representations with relation-aware dynamic parameter adaptation, so that candidate-specific relational information can influence both semantic representation and low-rank parameter updates, remains an important issue in knowledge graph-enhanced medical question answering.

### 2.2 Parameter efficient fine tuning and adaptive knowledge aware modeling

Parameter-efficient fine-tuning has become an important research direction in the field of large language models, with its core objective being to achieve effective adaptation to downstream tasks while updating as few parameters as possible. LoRA, proposed by Hu et al. [20], significantly reduced fine-tuning cost through low-rank matrix decomposition, laying the foundation for a large number of subsequent parameter-efficient methods. Building on this, Zhang et al. [21] further proposed AdaLoRA, which improves parameter utilization efficiency across different layers and modules through an adaptive budget allocation mechanism, indicating that fixed low-rank allocation is not necessarily suitable for complex task scenarios. As task heterogeneity increases, researchers have begun to introduce the mixture-of-experts paradigm into parameter-efficient fine-tuning. Luo et al. [22] improved the model’s representation capability in complex tasks through a contrastive learning-guided mixture-of-experts LoRA framework. Zhao et al. [23] further combined retrieval augmentation with multiple LoRA experts to strengthen the model’s dynamic ability to invoke external information. Yang et al. [24] and Zhang et al. [25] demonstrated, respectively from the perspectives of multi-task domain adaptation and adaptive composition of low-rank experts, that a single fixed adapter is often insufficient to balance sharedness and specificity in multi-relation, multi-task, or multi-domain environments. Overall, research on parameter-efficient fine-tuning has gradually evolved from the early unified low-rank update paradigm into a dynamic compositional adaptation framework oriented toward task differences and semantic heterogeneity. However, most existing methods still mainly perform adaptation selection based on overall task characteristics or retrieved information, while paying insufficient attention to finer-grained knowledge relational patterns within individual samples.

Another important research line that has developed in parallel with parameter-efficient fine-tuning is knowledge-aware modeling, which focuses on how to enable models to make more effective use of external knowledge and task semantic structures during the adaptation process. KnowTuning, proposed by Lyu et al. [26], showed that knowledge-aware fine-tuning can enhance the adaptability of large language models to knowledge-intensive tasks through explicit modeling of knowledge relevance, providing direct evidence for the collaborative modeling of knowledge and parameter updates. In the medical domain, Brzek et al. [27] achieved efficient medical question answering by combining QLoRA fine-tuning with knowledge distillation, indicating that parameter-efficient techniques have strong practical value in medical scenarios. He et al. [28] further extended parameter-efficient fine-tuning to multimodal medical settings, reflecting the strong cross-task scalability of this paradigm. Furthermore, Liu et al. [29] integrated large language model-driven medical knowledge retrieval with question answering at the system framework level, showing that knowledge enhancement is no longer limited to input-side supplementation, but is gradually shifting toward a unified design spanning the entire process of retrieval, representation, and generation. However, most existing knowledge-aware methods still adopt relatively coarse-grained knowledge injection or unified adaptation strategies, often lacking explicit differentiation of the dominant relation types across different samples, and rarely considering the construction of differentiated low-rank update pathways for heterogeneous medical relations such as symptom-disease, drug-disease, and disease-test. As a result, when facing complex medical multiple-choice questions, the model is prone to representation conflicts caused by different relation patterns sharing the same parameter update direction. Therefore, how to further couple knowledge relation identification mechanisms with dynamic parameter-efficient fine-tuning, and construct a knowledge-aware adaptation framework with sample-level relation pattern-adaptive activation, remains a key gap in current related research.

## 3 Methodology

### 3.1 Overall model architecture

This paper proposes a knowledge-enhanced large language model framework for medical multiple-choice question answering. Its overall architecture consists of an external medical knowledge guidance module, an option-level knowledge subgraph representation module, a pre-trained large language model semantic encoding module, and a relation-aware dynamic adaptation module. For a given medical question, the question stem and each candidate option are first subjected to medical entity recognition and concept linking, and local knowledge subgraphs corresponding to each candidate answer are extracted from external medical knowledge sources. Subsequently, a lightweight graph neural network is used to structurally encode the knowledge subgraphs corresponding to different candidate options, yielding information representations constrained by medical relations. Meanwhile, the question stem and each candidate option text are fed into a pre-trained large language model to obtain their corresponding semantic representations. In order to enable effective collaboration between structured knowledge and linguistic semantics in a unified space, the model maps the graph encoding results into the representation space of the large language model and fuses them with the semantic representations of the candidate options, thereby forming enhanced candidate representations that simultaneously contain textual semantic cues and medical relational information. The overall architecture of the model is shown in Fig 1.

**Fig 1:**
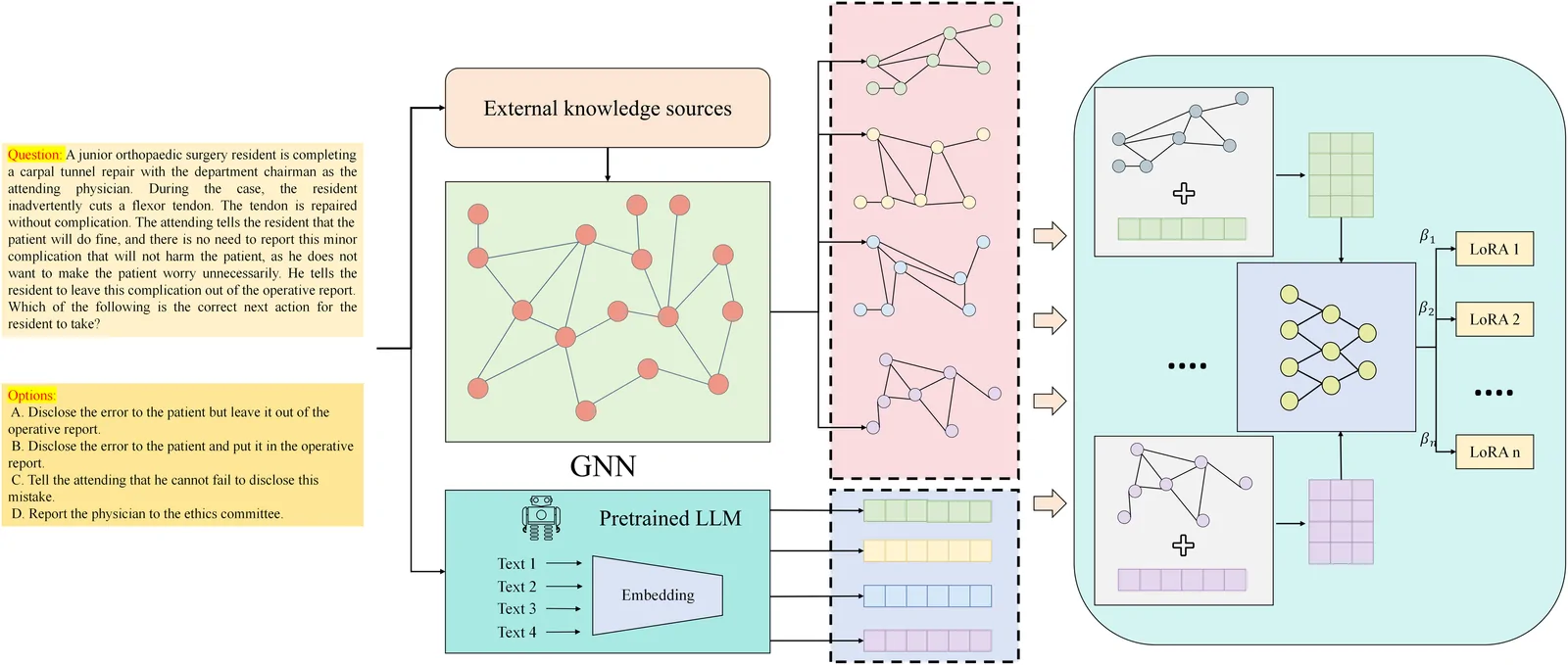
Overall model architecture.

On this basis, the model further introduces a relation-aware dynamic adaptation mechanism to generate candidate-conditioned parameter update paths from the relational information encoded in the enhanced candidate representations. Specifically, the fused representation of each candidate is processed by a relation-aware gating network to produce routing weights over multiple learned LoRA expert branches. The selected expert branches are dynamically combined to form a candidate-specific low-rank update for the post-fusion adaptation layer. In this way, different candidate options not only correspond to different local knowledge subgraphs, but can also activate differentiated combinations of learned low-rank experts according to their relational representations. Finally, the adapted representations of all candidate options are fed into a shared prediction head to output the corresponding scores, and the model determines the answer based on all candidate scores.

Let the input medical multiple-choice sample be denoted as


$$\begin{array}{c} 𝒳=\left(q,\{o_{i}{\}}_{i=1}^{N}\right) \end{array}$$ (1)

where *q* denotes the question stem, $o_{i}$ denotes the *i*-th candidate option, and *N* denotes the number of candidate answers. For each candidate option, a corresponding local knowledge subgraph $G_{i}$ is first constructed from external medical knowledge sources, and structural and semantic representations are extracted through a graph encoder and a pre-trained large language model, respectively. These are then fused in a unified space to obtain the enhanced candidate representation $h_{i}$, which can be formulated as


$$\begin{array}{c} h_{i}=Fusion\;\left(Proj\;\left(GNN(G_{i})\right),\;LLM(q,o_{i})\right),\;i=1,2,...,N \end{array}$$ (2)

After obtaining the enhanced candidate representations, the model employs a relation-aware gating network to generate dynamic routing weights over *C* learned LoRA expert branches. Let the relation pattern representation corresponding to the *i*-th candidate option be denoted as $r_{i}$, and let the *C* learned LoRA expert branches be denoted as $\{\Delta W_{c}{\}}_{c=1}^{C}$. The candidate-conditioned low-rank update can be expressed as


$$\begin{array}{c} \Delta W_{i}=\sum_{c=1}^{C}\alpha_{i,c}(r_{i})\Delta W_{c} \end{array}$$ (3)

where $\alpha_{i,c}(r_{i})$ denotes the routing weight assigned to the *c*-th learned LoRA expert branch according to the relation-aware representation $r_{i}$. The routing operation is performed after the backbone language-model forward pass and graph–text fusion, and the resulting low-rank update modulates a shared post-fusion adaptation matrix $W_{a}$. The adapted candidate representation and its prediction score are therefore written as


$$\begin{array}{c} {\tilde{h}}_{i}=\left(W_{a}+\lambda \Delta W_{i}\right)h_{i},\;s_{i}=Head\;\left({\tilde{h}}_{i}\right), \end{array}$$ (4)

where λ denotes the low-rank scaling coefficient, ${\tilde{h}}_{i}$ denotes the dynamically adapted representation of the *i*-th candidate option, and $s_{i}$ denotes its final prediction score. After normalizing and comparing the scores of all candidate options, the model outputs the option with the highest score as the final answer.

### 3.2 Option-level knowledge subgraph representation fusion

To model the fine-grained medical relations associated with different candidate options, this study adopts an option-level knowledge subgraph representation strategy. Building on previous LM–GNN approaches that construct local knowledge subgraphs for question–answer pairs [4–6], the proposed framework further uses the resulting candidate-specific relational representations as conditional information for subsequent relation-aware dynamic parameter adaptation.

For a given sample, the question stem and each candidate option are first subjected to entity recognition and concept normalization mapping, so that the entity set of the question stem and the entity sets of the candidate options can be obtained within a unified medical concept space.

A separate local knowledge subgraph is constructed for each candidate option, allowing the corresponding medical relation structures and reasoning clues to be preserved independently.

In the implementation, the external medical knowledge graph 𝒦 was instantiated using the 2024AB release of the Unified Medical Language System (UMLS) Metathesaurus. Medical entity mentions were detected using the en_core_sci_lg model provided by scispaCy, and the detected mentions were normalized to UMLS Concept Unique Identifiers using the scispaCy UMLS EntityLinker. For each mention, the highest-scoring candidate concept was retained when its linking score was not lower than 0.70; mentions without an eligible candidate were excluded from subsequent subgraph construction. To restrict graph expansion to clinically relevant terminology, UMLS concepts and relations were filtered by source vocabulary, retaining entries from SNOMEDCT_US, RXNORM, MSH, and LNC. For relational modeling, only MRREL edges whose REL field corresponded to PAR, CHD, RB, RN, or RO were retained. Fine-grained RELA labels were not modeled as independent edge types. This filtering reduces the influence of synonym-dominated and source-specific connections while preserving the principal hierarchical and related-concept structures used for local medical reasoning. Let the question stem be denoted as *q*, and let the candidate option set be denoted as $𝒪=\{o_{i}{\}}_{i=1}^{N}$. Then, the entity sets corresponding to the question stem and the *i*-th candidate option are respectively defined as


$$\begin{array}{c} {ℰ}_{q}=\Phi (q),\;{ℰ}_{i}=\Phi (o_{i}) \end{array}$$ (5)

where Φ(·) denotes the complete entity processing pipeline consisting of scispaCy-based medical mention detection, UMLS candidate generation, confidence-based candidate filtering, and mapping of the retained mention to its corresponding UMLS Concept Unique Identifier. Based on the joint results of the question entities and the candidate entities, the seed concept set corresponding to the *i*-th candidate option is constructed as


$$\begin{array}{c} {𝒮}_{i}={ℰ}_{q}\cup {ℰ}_{i} \end{array}$$ (6)

This design enables each candidate option to form a local knowledge entry point that is highly correlated with its own semantic content while sharing the contextual information of the same question stem, thereby providing a foundation for subsequent fine-grained structural modeling.

After the seed concept set is determined, a local knowledge subgraph is extracted from the filtered UMLS medical knowledge graph while explicitly constraining its expansion range and overall scale. Specifically, for the *i*-th candidate option, graph traversal starts from the retained seed concepts in ${𝒮}_{i}$ and retrieves their neighboring UMLS concepts and relations to obtain the local subgraph $G_{i}=(V_{i},R_{i},A_{i})$, where $V_{i}$, $R_{i}$, and $A_{i}$ denote the node set, relation set, and adjacency structure, respectively. The construction process is formalized as


$$\begin{array}{c} G_{i}=\Psi (𝒦,{𝒮}_{i},L) \end{array}$$ (7)

where Ψ(·) denotes the constrained subgraph extraction function and *L* denotes the maximum graph expansion depth. In all experiments, *L* was set to 2. The confidence threshold of 0.70 is applied exclusively during the preceding entity-linking stage to determine which text-linked UMLS concepts are eligible to enter the seed set ${𝒮}_{i}$. Starting from these retained seed concepts, breadth-first expansion is then performed for at most two hops over the filtered UMLS graph containing the five retained MRREL relation categories. Nodes introduced during graph expansion are obtained directly from the UMLS relational structure rather than from textual entity linking and therefore are not subjected to the entity-linking confidence threshold. To prevent excessive subgraph growth, each option-level subgraph is further limited to at most 128 nodes and 512 edges, with nodes prioritized according to their shortest-path distance from the seed concepts.

For each concept node $v\in V_{i}$, the corresponding UMLS preferred concept name $s_{v}$ is tokenized using the tokenizer of the backbone language model. The resulting tokens are mapped through the shared input embedding layer of the same backbone, and mean pooling is applied over the token-level embeddings to obtain the initial semantic representation


$$\begin{array}{c} e_{v}=MeanPool\;\left({Emb}_{LLM}\left(Tok(s_{v})\right)\right), \end{array}$$ (8)

where Tok(·) and ${Emb}_{LLM}(\cdot )$ denote the backbone tokenizer and its input embedding layer, respectively. The pooled representation is subsequently mapped to the graph feature space through a learnable linear projection,


$$\begin{array}{c} x_{v}=W_{x}e_{v}+b_{x}, \end{array}$$ (9)

where $x_{v}\in {\mathbb{R}}^{d_{g}}$ and $d_{g}=256$. The initial node feature matrix of the *i*-th candidate-specific subgraph is then constructed by stacking the projected representations of all nodes in $V_{i}$:


$$\begin{array}{c} X_{i}=[x_{v}{]}_{v\in V_{i}}\in {\mathbb{R}}^{|V_{i}|\times d_{g}}. \end{array}$$ (10)

This initialization directly reuses the semantic embedding space of the corresponding backbone language model without introducing an additional external biomedical embedding model.

Subsequently, a lightweight graph encoder is adopted to propagate and aggregate the initialized node representations over the candidate-specific relational structure. Specifically, a two-layer relational graph convolutional network (R-GCN) was used to model the five retained UMLS relation types, corresponding to PAR, CHD, RB, RN, and RO, with relation-specific transformation parameters. Each layer used a hidden dimension of 256, followed by ReLU activation and a dropout rate of 0.1. Let the initial node representation matrix of the *i*-th candidate subgraph be $X_{i}\in {\mathbb{R}}^{|V_{i}|\times d_{g}}$, and the graph-encoded structural representation is denoted as


$$\begin{array}{c} z_{i}=MeanPool\;\left(R\text{-}GCN(X_{i},A_{i},R_{i})\right) \end{array}$$ (11)

where R-GCN(·) denotes the two-layer relation-aware graph encoding process, and MeanPool(·) denotes mean aggregation over all node representations in the candidate-specific subgraph. For implementation convenience, the construction and fusion process of option-level knowledge subgraphs is executed according to Algorithm 1, which explicitly presents the complete procedure from entity extraction and subgraph construction to graph-text joint representation generation.

**Algorithm 1 Option-Level Knowledge Subgraph Representation Fusion Process**

**Require:** Question stem *q*, candidate option set $𝒪=\{o_{i}{\}}_{i=1}^{N}$, filtered UMLS medical knowledge graph 𝒦, scispaCy entity linker Φ, pre-trained language model LLM, two-layer five-relation relational graph convolutional network R-GCN

**Ensure:** Enhanced candidate representation set $ℋ=\{h_{i}{\}}_{i=1}^{N}$

1: Detect medical mentions in the question stem and link eligible mentions to UMLS concepts to obtain ${ℰ}_{q}\leftarrow \Phi (q)$

2: **for** *i* = 1 to *N* **do**

3:  Detect medical mentions in the *i*-th candidate option and link eligible mentions to UMLS concepts to obtain ${ℰ}_{i}\leftarrow \Phi (o_{i})$

4:  Construct the seed concept set ${𝒮}_{i}\leftarrow {ℰ}_{q}\cup {ℰ}_{i}$

5:  Extract the two-hop local knowledge subgraph $G_{i}\leftarrow \Psi (𝒦,{𝒮}_{i},2)$

6:  Obtain the graph structural representation $z_{i}\leftarrow MeanPool(R\text{-}GCN(X_{i},A_{i},R_{i}))$

7:  Use the pre-trained language model to obtain the textual semantic representation $t_{i}\leftarrow LLM(q,o_{i})$

8:  Map the graph representation into the language space ${\tilde{z}}_{i}\leftarrow W_{p}z_{i}+b_{p}$

9:  Fuse the graph representation and the textual representation to obtain the enhanced representation $h_{i}\leftarrow Fusion({\tilde{z}}_{i},t_{i})$

10: **end for**

11: Return $ℋ=\{h_{i}{\}}_{i=1}^{N}$

After obtaining the structural representation of the knowledge subgraph, it is still necessary to align it with the candidate textual semantic representation extracted by the large language model into the same representation space. Let the semantic representation output by the pre-trained large language model after jointly encoding the question stem and the *i*-th candidate option be denoted as $t_{i}\in {\mathbb{R}}^{d_{t}}$. Since the graph structural representation and the linguistic semantic representation are usually located in different feature spaces, the graph representation is first mapped into the language space through a linear projection, yielding


$$\begin{array}{c} {\tilde{z}}_{i}=W_{p}z_{i}+b_{p} \end{array}$$ (12)

where $W_{p}\in {\mathbb{R}}^{d_{t}\times d_{g}}$ and $b_{p}\in {\mathbb{R}}^{d_{t}}$ are learnable projection parameters. On this basis, this paper adopts a gated fusion strategy to jointly model graph structural semantics and textual contextual semantics, enabling the model to dynamically regulate the contributions of the two types of information according to the content of the current candidate option. Specifically, the fusion gate vector is defined as


$$\begin{array}{c} g_{i}=\sigma \;\left(W_{g}[\;t_{i};{\tilde{z}}_{i}\;]+b_{g}\right) \end{array}$$ (13)

where [·;·] denotes vector concatenation, σ(·) denotes the Sigmoid activation function, and $W_{g}$ and $b_{g}$ are the gating parameters. Based on this gating mechanism, the final enhanced candidate representation is defined as


$$\begin{array}{c} h_{i}=g_{i}⊙t_{i}+(1-g_{i})⊙{\tilde{z}}_{i} \end{array}$$ (14)

where ⊙ denotes element-wise multiplication. This design avoids the redundant noise introduced by simple concatenation, so that the relational structure in the knowledge graph supplements the candidate representation of the language model only along necessary dimensions, thereby improving the discriminability and medical consistency of candidate representations. Finally, the enhanced representations of all candidate options are fed into a shared prediction layer for answer discrimination, although this subsection focuses more on the representation construction process itself.

The candidate-specific subgraphs preserve local medical relation patterns associated with different answer choices, allowing differentiated connections among concepts such as symptoms, diseases, drugs, and tests to be retained in the representation space. The resulting graph-enhanced representations provide a structured basis for distinguishing candidate answers whose textual semantics may otherwise appear similar. In this way, the correct option is not only more consistent with the semantic meaning of the question stem at the textual level, but also forms more reasonable medical relational support with the entities in the question stem at the graph structural level, whereas distractor options often correspond to weaker or inconsistent local relational paths. Importantly, within the proposed framework, these candidate-specific relational representations are not used solely for answer representation enhancement. They are further passed to the subsequent relation-aware dynamic LoRA adaptation module as relation-pattern inputs, thereby linking structured knowledge modeling with dynamic parameter adaptation.

### 3.3 Relation-aware dynamic LoRA adaptation

After obtaining the knowledge-enhanced representations of the candidate options, the model further introduces a relation-aware dynamic LoRA adaptation mechanism to improve the adaptability of parameter-efficient fine-tuning in heterogeneous medical relation scenarios. Different from using a single fixed LoRA adapter to perform unified updates for all samples, this module extracts candidate-conditioned relational information from the fused representation derived from option-level knowledge subgraph modeling, and then dynamically weights multiple learned LoRA expert branches through a relation-aware gating network, thereby generating differentiated low-rank update paths for different candidate representations. Since the enhanced candidate representation constructed in the previous subsection has already explicitly integrated the question semantics, option semantics, and their local knowledge subgraph relations, this representation can be used not only for answer discrimination but also as a direct input for relation pattern identification. The learned LoRA expert branches in RADA are attached to a shared post-fusion linear adaptation layer operating on the enhanced candidate representation $h_{i}$, rather than to the internal Transformer projection layers of the backbone language model. Accordingly, the backbone language-model forward pass and graph–text fusion are completed before the routing weights are computed, after which the selected low-rank expert branches modulate the post-fusion adaptation layer. The architecture of this module is illustrated in Fig 2.

**Fig 2:**
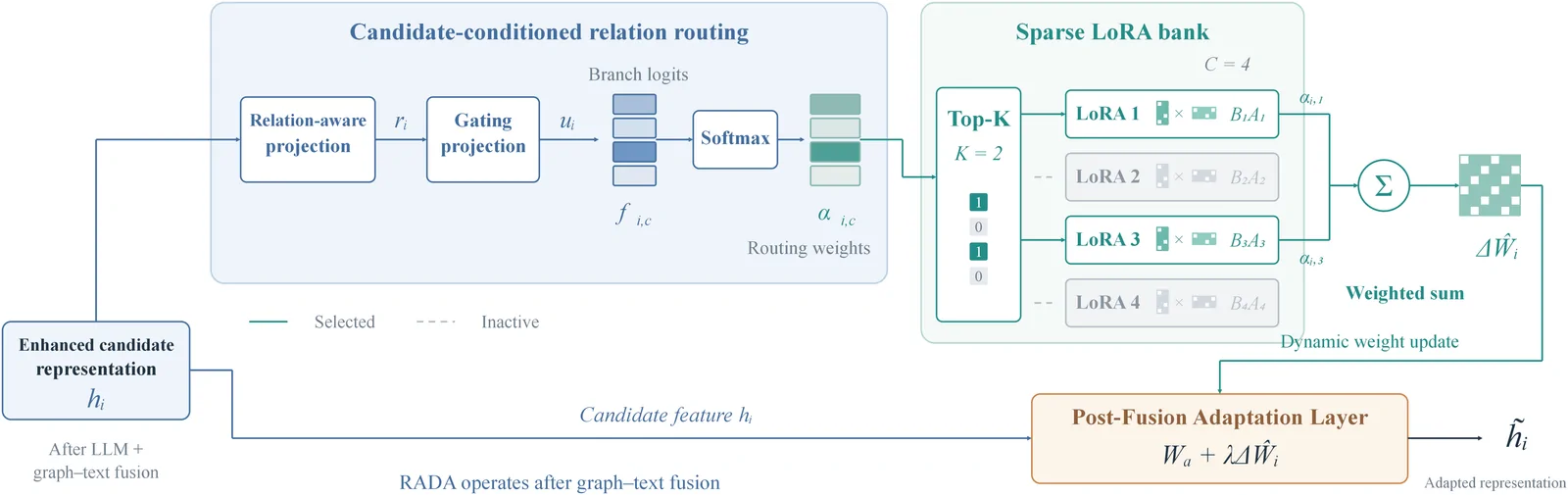
The RADA module extracts relation-aware features from the enhanced candidate representations, generates routing weights over four learned LoRA expert branches, and selects the most relevant top-*K* experts to construct a candidate-specific low-rank update for the post-fusion adaptation layer.

Let the fused representation corresponding to the *i*-th candidate option be denoted as $h_{i}\in {\mathbb{R}}^{d}$. A relation-aware projection network is first used to extract its relation pattern features:


$$\begin{array}{c} r_{i}=\phi (W_{r}h_{i}+b_{r}) \end{array}$$ (15)

where $W_{r}$ and $b_{r}$ are learnable parameters, ϕ(·) denotes a nonlinear transformation function, and $r_{i}$ denotes the relation pattern representation of the *i*-th candidate option. To further compress redundant information and highlight dominant relational characteristics, the relation-gated representation is defined as


$$\begin{array}{c} u_{i}=\psi (W_{u}r_{i}+b_{u}) \end{array}$$ (16)

where $W_{u}$ and $b_{u}$ denote the gating projection parameters, and ψ(·) denotes an activation function. This process enables the model to extract a more compact and discriminative relation-aware vector from the enhanced candidate representation, thereby providing conditional constraints for subsequent dynamic adaptation.

After obtaining the relation-aware vector, the model uses a lightweight gating network to generate combination weights over multiple learned LoRA expert branches. Assume that *C* learnable LoRA expert branches are defined in the model. These branches are not explicitly assigned to predefined medical relation categories or individual UMLS relation types; instead, their specialization is learned end-to-end through candidate-conditioned routing during training. In all experiments, the number of LoRA expert branches is fixed to *C* = 4. Then, the unnormalized response of the *i*-th candidate option to the *c*-th branch is defined as


$$\begin{array}{c} f_{i,c}=w_{c}^{⊤}u_{i}+b_{c},\;c=1,2,...,C \end{array}$$ (17)

where $w_{c}$ and $b_{c}$ denote the gating parameters associated with the *c*-th LoRA expert branch. In order to transform the responses of different branches into comparable adaptation strengths, Softmax normalization is applied to obtain the dynamic combination weights of all branches:


$$\begin{array}{c} \alpha_{i,c}=\frac{\exp(f_{i,c})}{\sum_{j=1}^{C}\exp(f_{i,j})} \end{array}$$ (18)

where $\alpha_{i,c}$ denotes the activation coefficient of the *c*-th learned LoRA expert branch for the *i*-th candidate option. This mechanism implies that the model no longer shares a single low-rank update direction for all samples, but instead adaptively adjusts the contribution of each learned expert according to the candidate-specific relational representation, thereby allowing different candidates to activate differentiated low-rank update paths.

For the shared post-fusion adaptation matrix $W_{a}\in {\mathbb{R}}^{d_{out}\times d_{in}}$, the *c*-th learned LoRA expert branch adopts the standard low-rank decomposition form to construct its parameter increment:


$$\begin{array}{c} \Delta W_{c}=B_{c}A_{c} \end{array}$$ (19)

where $A_{c}\in {\mathbb{R}}^{r\times d_{in}}$, $B_{c}\in {\mathbb{R}}^{d_{out}\times r}$, and *r* denotes the low-rank dimension. Based on the dynamic combination weights of all branches, the adaptive low-rank update corresponding to the *i*-th candidate option is defined as


$$\begin{array}{c} \Delta W_{i}=\sum_{c=1}^{C}\alpha_{i,c}\Delta W_{c} \end{array}$$ (20)

Furthermore, considering that only a subset of learned experts may be strongly activated for a given candidate representation, this paper retains only the top-*K* LoRA expert branches with the highest routing weights during the dynamic fusion stage, thereby suppressing contributions from weakly activated branches. Let ${𝒯}_{i}=TopK(\{\alpha_{i,c}{\}}_{c=1}^{C},K)$ denote the index set of the top-*K* activated relation branches for the *i*-th candidate option. Then, the sparsified dynamic update can be written as


$$\begin{array}{c} \Delta {\hat{W}}_{i}=\sum_{c\in {𝒯}_{i}}\alpha_{i,c}\Delta W_{c} \end{array}$$ (21)

This design is consistent with the expert selection process shown in the module architecture, enabling the model to make more concentrated use of the learned update paths that receive the strongest candidate-conditioned routing weights while maintaining parameter efficiency.

Finally, the shared post-fusion adaptation matrix and the relation-aware dynamic low-rank update jointly transform the enhanced candidate representation. Since the routing coefficients are obtained from the already-computed representation $h_{i}$, this operation is performed after the backbone language-model forward pass and graph–text fusion. The adapted representation is defined as


$$\begin{array}{c} {\tilde{h}}_{i}=(W_{a}+\lambda \Delta {\hat{W}}_{i})h_{i} \end{array}$$ (22)

where $W_{a}$ denotes the shared post-fusion adaptation matrix, λ denotes the scaling coefficient of the low-rank update, and ${\tilde{h}}_{i}$ denotes the final representation of the *i*-th candidate option after relation-aware dynamic adaptation. Compared with a fixed low-rank adaptation applied to the same post-fusion transformation, RADA dynamically reorganizes the low-rank update path at the candidate level according to the relational information encoded in the candidate representation. This allows different candidate representations to activate different combinations of learned low-rank expert subspaces while leaving the internal Transformer computation of the backbone language model unaffected. In this way, the option-level knowledge subgraph fusion representation constructed in the previous subsection not only enhances the separability of medical relations in candidate representations but also provides the routing signal required for subsequent post-fusion dynamic adaptation.

### 3.4 Loss function

The proposed framework is trained end-to-end using a candidate-level classification objective together with a lightweight load-balancing regularization for the relation-aware routing network. For each question, the dynamically adapted representation ${\tilde{h}}_{i}$ of the *i*-th candidate is mapped to a scalar score,


$$\begin{array}{c} s_{i}=Head({\tilde{h}}_{i}), \end{array}$$ (23)

and the probability of selecting candidate *i* is defined as


$$\begin{array}{c} p_{i}=\frac{\exp(s_{i})}{\sum_{j=1}^{N}\exp(s_{j})}. \end{array}$$ (24)

Given a mini-batch of size *B*, the primary medical question-answering loss is the multi-class cross-entropy


$$\begin{array}{c} {ℒ}_{QA}=-\frac{1}{B}\sum_{b=1}^{B}\log p_{b,y_{b}}, \end{array}$$ (25)

where $y_{b}$ denotes the correct candidate index of the *b*-th question.

To prevent the relation-aware gating network from being persistently dominated by a small subset of LoRA expert branches, a lightweight load-balancing term is introduced. Let *M* denote the total number of candidate representations in the current mini-batch, ${𝒯}_{m}$ denote the Top-*K* selected branch set for the *m*-th candidate, and $\alpha_{m,c}$ denote the soft routing weight of branch *c*. The branch utilization frequency and average routing probability are defined as


$$\begin{array}{c} f_{c}=\frac{1}{MK}\sum_{m=1}^{M}𝕀\;\left(c\in {𝒯}_{m}\right),\;P_{c}=\frac{1}{M}\sum_{m=1}^{M}\alpha_{m,c}. \end{array}$$ (26)

The corresponding load-balancing loss is


$$\begin{array}{c} {ℒ}_{bal}=C\sum_{c=1}^{C}f_{c}P_{c}. \end{array}$$ (27)

The final training objective is therefore


$$\begin{array}{c} ℒ={ℒ}_{QA}+\lambda_{bal}{ℒ}_{bal}, \end{array}$$ (28)

where $\lambda_{bal}=0.01$ controls the contribution of the routing regularization.

## 4 Experimental results and analysis

### 4.1 Dataset introduction

#### 4.1.1 MedQA.

MedQA [30] is one of the representative multiple-choice datasets in the field of medical question answering. Its questions are mainly derived from medical licensing examinations and therefore exhibit strong domain specificity and clinical reasoning characteristics. The dataset covers three language versions, including English, Simplified Chinese, and Traditional Chinese, among which the English subset is commonly used for the evaluation of medical large language models on multiple-choice question answering tasks. Since this study conducts experiments in the setting of English medical multiple-choice questions, the English subset of MedQA is adopted as one of the benchmark datasets. Each sample in this dataset contains a question stem, candidate options, and a gold answer, making it suitable for evaluating the overall capability of the model in medical knowledge understanding, relation discrimination, and answer prediction.

#### 4.1.2 MedMCQA.

MedMCQA [31] is a large-scale benchmark dataset for multiple-choice question answering in the medical domain. Its questions are mainly derived from real medical entrance examinations and professional assessment materials, including AIIMS and NEET PG examinations, and therefore exhibit broad medical knowledge coverage and a high density of professional terminology. Each sample contains a question stem, four candidate options, the correct-option annotation, and, where available, an expert explanation. The dataset covers 21 medical subjects and approximately 2,400 healthcare topics, including anatomy, biochemistry, medicine, pathology, pharmacology, physiology, and surgery.

The original MedMCQA dataset contains 182,822 training questions, 4,183 validation questions, and 6,150 test questions. Since the answer labels of the official test set are not publicly available, the official test split was not used for performance evaluation in this study. Instead, 7,000 questions were randomly held out from the original training split to construct the validation set used in the experiments. The remaining 175,822 questions were used for model training. The held-out validation set was used for model selection and hyperparameter tuning, while the official validation set containing 4,183 questions was used as the test set for performance evaluation. The resulting experimental split used in this study is summarized in Table 1.

**Table 1:** Statistics of the MedMCQA split used in this study.

Statistic	Value
Original training questions	182,822
Training questions used in this study	175,822
Randomly held-out validation questions	7,000
Test questions (official validation set)	4,183
Official test questions (labels unavailable)	6,150

### 4.2 Experimental setup

All experiments were conducted under a unified software and hardware environment to ensure the comparability of results across different models and datasets. Specifically, Llama3 8B and Qwen 7B were selected as the backbone large language models, and training and evaluation were performed on the MedQA and MedMCQA datasets. The training platform consisted of four NVIDIA H100 GPUs, which provided sufficient computational resources for parameter-efficient fine-tuning, knowledge subgraph encoding, and batch inference in large-scale medical multiple-choice question answering tasks. During training, the AdamW optimizer was adopted for parameter updates, together with a linear warmup and cosine decay schedule to stabilize the optimization process. Meanwhile, in order to balance GPU memory usage and training efficiency, mixed-precision training was employed, and parallel training and inference were conducted in a multi-GPU environment. The same maximum input length, batch size, learning rate, and low-rank adaptation scale were consistently used during model training to reduce the interference of additional variables on the results. To reduce potential answer-position bias, the candidate options were randomly permuted before evaluation, with the corresponding gold labels remapped consistently according to the same permutation. The main software and hardware configurations and the core hyperparameter settings used in this study are shown in Table 2.

**Table 2:** Software and hardware environment and main hyperparameter settings.

Item	Setting
Backbone Model 1	meta-llama/Meta-Llama-3-8B-Instruct
Backbone Model 2	Qwen/Qwen1.5-7B-Chat
Number of GPUs	4
GPU Type	NVIDIA H100
Computation Precision	BF16
Deep Learning Framework	PyTorch
CUDA Version	12.1
Optimizer	AdamW
Initial Learning Rate	$2\times 10^{-4}$
Weight Decay	0.01
Learning Rate Schedule	Linear Warmup + Cosine Decay
Warmup Ratio	0.03
Number of Training Epochs	5
Batch Size per GPU	8
Total Effective Batch Size	32
Gradient Accumulation Steps	1
Maximum Input Length	1024
Maximum Output Length	128
LoRA Rank *r*	16
LoRA α	32
LoRA Dropout	0.05
Number of Top-*k* Relation Branches	2
Number of Relation-Specific LoRA Branches *C*	4
Number of Graph Encoder Layers	2
Graph Representation Dimension $d_{g}$	256
Text Representation Dimension $d_{t}$	4096
Decoding Strategy	Sampling
Do Sample	True
Temperature	0.7
Top-p	0.9
Number of Independent Runs	3

For the AMG-RAG baseline, we reimplemented the method under the same evaluation setting used in this study and evaluated it with Llama3 8B and Qwen 7B as the backbone models. The original retrieval-based reasoning procedure of AMG-RAG was retained, and the method was evaluated in a training-free setting without additional parameter fine-tuning. The same implementation and evaluation protocol was applied to both MedQA and MedMCQA. The AMG-RAG results reported in the comparative experiments were obtained from our own reimplementation rather than directly copied from the original publication.

For the federated LoRA-based baselines, including FedLoRA and LoRA-FAIR, we adopted a centralized adaptation setting because the present study does not involve distributed clients or federated data partitions. Specifically, all training samples were processed within a single centralized training pipeline, while the low-rank adaptation components and optimization strategies of the corresponding methods were retained. Client partitioning, inter-client communication, and federated parameter aggregation were not performed. Therefore, these methods were included as alternative low-rank adaptation baselines rather than as federated learning frameworks. This setting enables a direct comparison of different parameter-efficient adaptation strategies under the same backbone models, datasets, and evaluation protocol.

### 4.3 Evaluation metric

To comprehensively evaluate the discriminative performance of the model on medical multiple-choice question answering tasks, this study adopts Accuracy as the primary evaluation metric and additionally reports macro-averaged Precision, Recall, and F1-score. Since each question contains multiple candidate options but only one gold answer, the task is formulated as a single-label multi-class classification problem. Accuracy directly measures the proportion of questions for which the model selects the correct option, while macro-averaged Precision, Recall, and F1-score evaluate the prediction performance across different answer-option classes by assigning equal importance to each class.

Let *N* denote the total number of questions, $y_{i}$ denote the gold answer label of the *i*-th question, and ${\hat{y}}_{i}$ denote the corresponding predicted answer label. Accuracy is defined as


$$\begin{array}{c} Accuracy=\frac{1}{N}\sum_{i=1}^{N}𝕀\;\left({\hat{y}}_{i}=y_{i}\right), \end{array}$$ (29)

where 𝕀(·) denotes the indicator function.

For each answer-option class *c*, true positives, false positives, and false negatives are denoted as $TP_{c}$, $FP_{c}$, and $FN_{c}$, respectively. The class-wise Precision and Recall are calculated as


$$\begin{array}{c} {Precision}_{c}=\frac{TP_{c}}{TP_{c}+FP_{c}}, \end{array}$$ (30)

$$\begin{array}{c} {Recall}_{c}=\frac{TP_{c}}{TP_{c}+FN_{c}}. \end{array}$$ (31)

The class-wise F1-score is defined as


$$\begin{array}{c} {F1}_{c}=\frac{2\times {Precision}_{c}\times {Recall}_{c}}{{Precision}_{c}+{Recall}_{c}}. \end{array}$$ (32)

Let *C* denote the number of answer-option classes. The macro-averaged Precision, Recall, and F1-score are then calculated as


$$\begin{array}{c} Macro\text{-}Precision=\frac{1}{C}\sum_{c=1}^{C}{Precision}_{c}, \end{array}$$ (33)

$$\begin{array}{c} Macro\text{-}Recall=\frac{1}{C}\sum_{c=1}^{C}{Recall}_{c}, \end{array}$$ (34)

$$\begin{array}{c} Macro\text{-}F1=\frac{1}{C}\sum_{c=1}^{C}{F1}_{c}. \end{array}$$ (35)

In the medical multiple-choice question answering setting, Accuracy provides the most direct measure of whether the model selects the correct answer and is therefore treated as the primary evaluation metric. Macro-Precision, Macro-Recall, and Macro-F1 provide complementary information by evaluating predictive performance across answer-option classes without weighting the metrics by class frequency. The joint use of these metrics provides a more comprehensive assessment of the model’s candidate discrimination and answer prediction performance.

### 4.4 Experimental results compared with other models

To evaluate the overall performance of the proposed framework on medical multiple-choice question answering, we compare it with representative parameter-efficient fine-tuning, knowledge-enhanced reasoning, and full fine-tuning methods under the same experimental setting. The comparison includes FedLoRA, Med-REFL, LoftQ, HydraLoRA, LoRA-FAIR, AMG-RAG, GreaseLM, Towards Specialized Generalists, and Full Fine-Tuning. Although FedLoRA and LoRA-FAIR were originally developed in federated learning settings, their centralized implementations are used here as alternative low-rank adaptation baselines so that different adaptation strategies can be compared under the same backbone models and evaluation protocol. All methods are evaluated using Llama3 8B and Qwen 7B as backbone models, and the results are reported as the mean and standard deviation over three independent random-seed runs. The experimental results on MedQA are presented in Table 3.

**Table 3:** Experimental results compared with other models on MedQA, reported as the mean and standard deviation over three random-seed runs.

Backbone	Method	Acc	Precision	Recall	F1-Score
Llama3 8B	FedLoRA [32]	58.58±0.52	58.83±1.18	58.45±0.61	58.19±0.62
	Med-REFL [33]	60.54±1.14	61.31±0.88	60.53±1.02	60.37±0.98
	LoftQ [34]	59.49±1.01	60.31±1.21	59.40±0.97	59.47±0.83
	HydraLoRA [35]	61.59±0.48	61.51±0.98	61.49±0.48	61.03±0.86
	LoRA-FAIR [36]	59.33±0.68	58.87±0.65	59.42±0.72	58.99±0.55
	AMG-RAG [18]	61.40±0.79	62.01±0.96	61.29±0.75	60.91±0.48
	GreaseLM [5]	61.95±0.71	62.19±0.86	61.86±0.71	61.90±0.74
	Towards Specialized Generalists [24]	61.64±1.00	61.79±0.87	61.54±0.96	61.33±1.19
	Full Fine-Tuning	61.59±0.83	61.81±0.82	61.55±0.81	61.46±0.80
	Ours	** 62.90±0.82 **	** 63.57±1.22 **	** 62.86±0.90 **	** 62.97±1.07 **
Qwen 7B	FedLoRA	58.81±0.47	59.61±1.21	58.76±0.58	58.65±0.86
	Med-REFL	59.47±0.36	59.83±0.51	59.58±0.37	59.02±0.59
	LoftQ	59.94±0.96	61.70±1.09	59.89±0.97	60.51±0.88
	HydraLoRA	59.13±0.47	59.06±0.91	59.20±0.37	59.01±0.79
	LoRA-FAIR	59.94±0.59	61.07±0.72	59.91±0.68	60.26±0.73
	AMG-RAG	61.09±0.64	61.46±1.36	61.00±0.72	60.91±1.15
	GreaseLM	61.82±0.68	61.99±0.79	61.73±0.71	61.68±0.69
	Towards Specialized Generalists	61.59±0.79	61.43±1.03	61.49±0.82	61.08±1.05
	Full Fine-Tuning	61.64±0.71	61.89±0.74	61.63±0.71	61.55±0.72
	Ours	** 62.84±1.04 **	** 63.36±0.72 **	** 62.74±1.01 **	** 62.79±0.78 **

As shown in Table 3, the proposed method achieves the highest mean Accuracy, Precision, Recall, and F1-Score under both backbone models. With Llama3 8B, the proposed framework obtains an Accuracy of 62.90±0.82, a Precision of 63.57±1.22, a Recall of 62.86±0.90, and an F1-Score of 62.97±1.07. With Qwen 7B, the corresponding results are 62.84±1.04, 63.36±0.72, 62.74±1.01, and 62.79±0.78, respectively. The comparative results show that methods incorporating structured knowledge or adaptive parameter-efficient tuning generally achieve competitive performance, while the proposed framework consistently maintains strong results across all four evaluation metrics. This pattern is observed for both Llama3 8B and Qwen 7B, suggesting that combining option-level knowledge subgraph representations with candidate-conditioned dynamic low-rank adaptation provides a consistent contribution to candidate discrimination and answer prediction under the evaluated MedQA settings.

To further evaluate the performance of the proposed framework under a larger-scale medical question answering setting, we conduct comparative experiments on the MedMCQA dataset using the same two backbone models, Llama3 8B and Qwen 7B. The comparison includes representative parameter-efficient fine-tuning methods, knowledge-enhanced approaches, and full fine-tuning. All methods are evaluated under the same experimental protocol, and the results are reported as the mean and standard deviation over three independent random-seed runs. The comparative results on MedMCQA are presented in Table 4.

**Table 4:** Experimental results compared with other models on MedMCQA, reported as the mean and standard deviation over three random-seed runs.

Backbone	Method	Acc	Precision	Recall	F1-Score
Llama3 8B	FedLoRA	57.57±0.41	56.76±1.32	56.61±0.63	56.63±0.61
	Med-REFL	59.22±0.75	59.18±0.57	58.54±1.00	58.82±0.72
	LoftQ	57.37±0.98	57.87±0.81	55.45±1.01	56.34±0.32
	HydraLoRA	58.22±1.04	57.97±0.81	59.05±0.36	58.41±0.34
	LoRA-FAIR	57.34±1.34	57.19±1.08	58.67±0.37	57.23±0.47
	AMG-RAG	59.47±0.82	60.42±1.15	59.36±0.31	59.83±0.60
	GreaseLM	60.12±0.69	60.54±0.77	59.87±0.64	60.18±0.66
	Towards Specialized Generalists	59.70±0.80	59.76±1.14	59.19±0.39	59.01±0.45
	Full Fine-Tuning	59.81±0.72	59.83±0.79	59.26±0.70	59.45±0.66
	Ours	** 61.04±0.30 **	** 62.19±1.26 **	** 61.16±1.37 **	** 61.21±0.54 **
Qwen 7B	FedLoRA	54.69±1.38	54.37±0.68	55.03±0.83	54.47±0.23
	Med-REFL	55.41±0.48	55.74±0.58	54.67±1.26	55.04±0.56
	LoftQ	54.21±0.68	54.59±1.36	52.84±1.18	53.45±0.92
	HydraLoRA	56.20±1.33	55.09±0.41	55.73±0.57	55.38±0.46
	LoRA-FAIR	54.98±0.30	54.50±0.59	54.05±0.65	54.24±0.32
	AMG-RAG	57.26±0.35	57.30±1.14	56.55±0.33	56.54±0.62
	GreaseLM	57.64±0.62	57.88±0.69	57.32±0.59	57.53±0.16
	Towards Specialized Generalists	56.74±1.10	56.74±0.58	55.69±0.37	56.17±0.46
	Full Fine-Tuning	57.28±0.83	56.44±0.70	56.70±0.66	56.41±0.61
	Ours	** 58.59±0.65 **	** 59.06±0.44 **	** 57.99±0.58 **	** 57.77±0.57 **

As shown in Table 4, the proposed method obtains the highest mean values for Accuracy, Precision, Recall, and F1-Score under both backbone models. With Llama3 8B, the proposed framework achieves an Accuracy of 61.04±0.30, a Precision of 62.19±1.26, a Recall of 61.16±1.37, and an F1-Score of 61.21±0.54. Among the comparison methods, GreaseLM provides the closest overall results, with an Accuracy of 60.12±0.69 and an F1-Score of 60.18±0.66. A similar pattern is observed with Qwen 7B, where the proposed method reaches 58.59±0.65 Accuracy, 59.06±0.44 Precision, 57.99±0.58 Recall, and 57.77±0.57 F1-Score. These results are consistently higher than those obtained by the corresponding comparison methods across the four reported metrics. The results under both backbone models indicate that jointly incorporating option-level knowledge subgraph representations and candidate-conditioned dynamic low-rank adaptation provides consistent improvements in medical candidate discrimination and answer prediction on MedMCQA.

To further examine whether the observed Accuracy differences are consistent at the test-item level, paired bootstrap analyses were conducted between the proposed method and four representative comparison methods using 10,000 bootstrap resamples. The analysis was performed separately for each dataset–backbone configuration, and the resulting two-sided *p*-values were adjusted for multiple comparisons using the Holm procedure.

As shown in Fig 3, all paired Accuracy differences favor the proposed method across the evaluated dataset–backbone configurations. The bootstrap analysis provides further statistical support for the comparative results, with the evidence being particularly consistent on MedMCQA. Under Llama3 8B, the performance gains over AMG-RAG and HydraLoRA remain significant after Holm correction, while under Qwen 7B, all four comparisons retain statistical significance. On MedQA, the statistical evidence is comparatively weaker, although the improvement over HydraLoRA under Qwen 7B remains significant after correction. Taken together, these results provide additional support for the robustness of the observed performance gains while showing that their statistical strength differs across evaluation settings.

**Fig 3:**
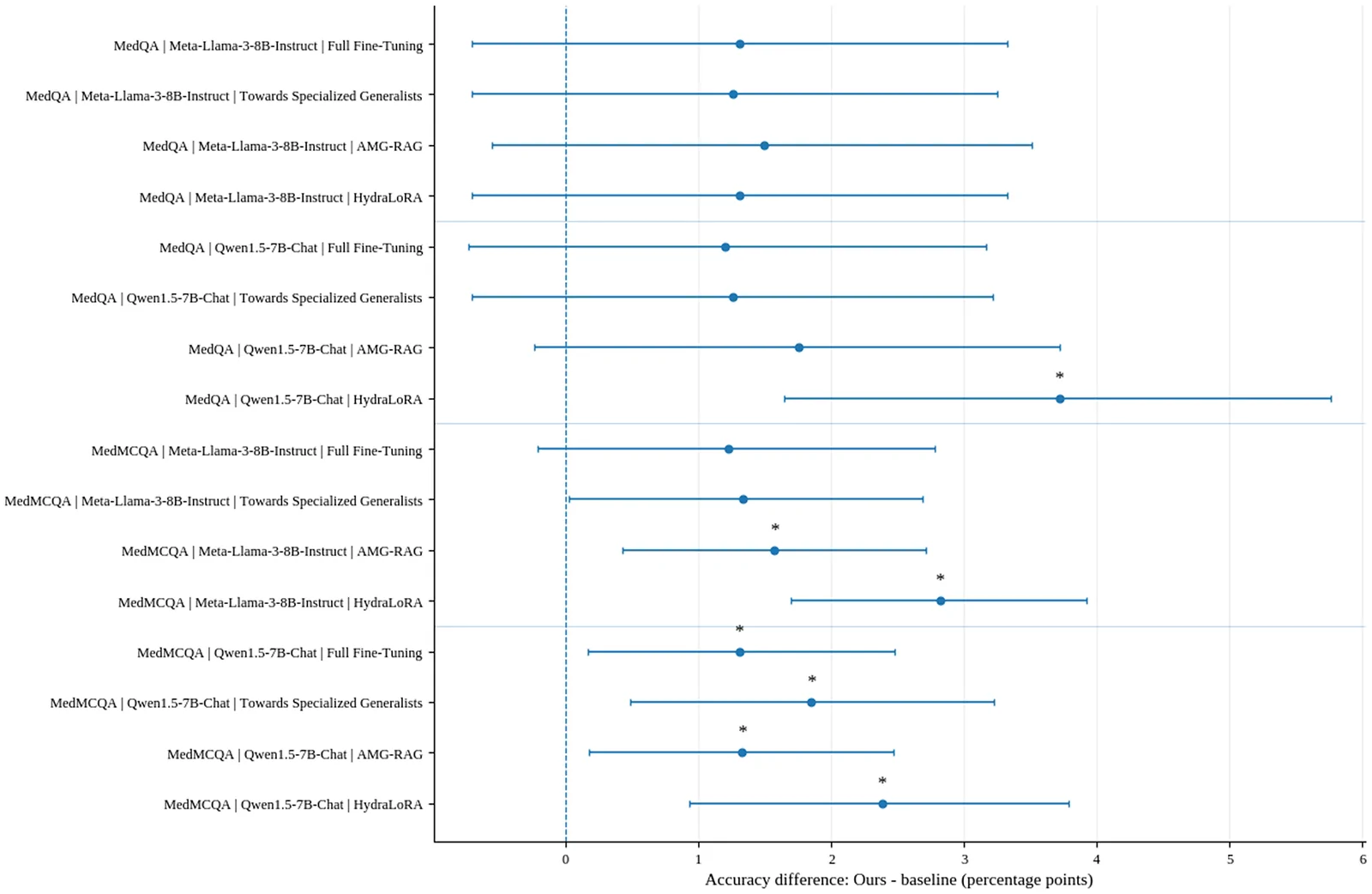
Paired bootstrap analysis of Accuracy differences between the proposed method and four comparison methods on MedQA and MedMCQA. Error bars denote 95% confidence intervals from 10,000 resamples, and asterisks indicate significance after Holm correction (*p*_Holm_ < 0.05).

### 4.5 Ablation test results

To examine the contribution of the main components of the proposed framework, ablation experiments were conducted on both MedQA and MedMCQA using Llama3 8B and Qwen 7B as backbone models. The experiments compare zero-shot and few-shot backbone settings, conventional full fine-tuning, standard LoRA, LoRA combined separately with the Option-Level Knowledge Subgraph (OLKS) module and the Relation-Aware Dynamic LoRA Adaptation (RADA) module, and the complete framework integrating both modules. All configurations are evaluated using the same datasets and evaluation metrics, and the results are reported as the mean and standard deviation over three independent runs. The complete ablation results are presented in Table 5.

**Table 5:** Ablation test results on MedQA and MedMCQA, reported as the mean and standard deviation over three random-seed runs.

Dataset	Backbone	Method	Acc	Precision	Recall	F1-Score
MedQA	Llama3 8B	Backbone (Zero-shot)	49.59±0.92	49.83±0.88	49.27±0.74	49.17±0.78
		Backbone (3-shot)	52.13±0.78	52.69±0.78	51.73±0.64	52.05±0.73
		Full Fine-Tuning	61.59±0.83	61.81±0.82	61.55±0.81	61.46±0.80
		LoRA	58.65±0.66	59.28±0.42	57.58±0.69	58.00±0.48
		+OLKS	60.59±0.28	61.37±1.44	59.62±0.33	60.16±0.66
		+RADA	61.40±1.23	61.41±1.15	60.96±0.68	60.98±0.98
		Ours	** 62.90±0.82 **	** 63.57±1.22 **	** 62.86±0.90 **	** 62.97±1.07 **
	Qwen 7B	Backbone (Zero-shot)	57.97±1.23	58.40±0.50	57.77±0.99	57.79±0.65
		Backbone (3-shot)	59.13±0.73	59.54±0.75	58.81±0.71	58.88±0.51
		Full Fine-Tuning	61.64±0.71	61.89±0.74	61.63±0.71	61.55±0.72
		LoRA	57.32±0.91	57.78±0.34	56.89±0.85	56.93±0.24
		+OLKS	60.49±1.14	60.36±0.62	59.72±0.37	59.93±0.40
		+RADA	59.86±0.52	60.38±0.36	58.89±1.15	59.08±0.61
		Ours	** 62.84±1.04 **	** 63.36±0.72 **	** 62.74±1.01 **	** 62.79±0.78 **
MedMCQA	Llama3 8B	Backbone (Zero-shot)	47.70±0.57	48.06±1.09	47.10±0.80	47.04±0.43
		Backbone (3-shot)	51.63±0.81	52.06±0.86	51.10±0.74	51.43±0.85
		Full Fine-Tuning	59.81±0.72	59.83±0.79	59.26±0.70	59.45±0.66
		LoRA	57.10±1.03	56.81±0.40	56.34±1.25	56.30±0.93
		+OLKS	58.91±0.67	58.78±0.77	58.42±0.70	58.46±0.71
		+RADA	59.68±1.09	59.42±0.22	59.13±0.93	59.19±0.56
		Ours	** 61.04±0.30 **	** 62.19±1.26 **	** 61.16±1.37 **	** 61.21±0.54 **
	Qwen 7B	Backbone (Zero-shot)	54.98±0.88	55.73±0.81	54.99±1.08	55.08±0.78
		Backbone (3-shot)	56.18±0.61	56.57±0.71	55.71±0.58	56.01±0.54
		Full Fine-Tuning	57.28±0.83	56.44±0.70	56.70±0.66	56.41±0.61
		LoRA	55.04±0.90	55.53±0.48	54.14±0.73	54.62±0.66
		+OLKS	56.75±1.21	56.93±0.79	56.22±1.35	56.31±1.02
		+RADA	55.96±1.06	56.84±0.38	55.30±0.33	55.76±0.37
		Ours	** 58.59±0.65 **	** 59.06±0.44 **	** 57.99±0.58 **	** 57.77±0.57 **

As shown in Table 5, few-shot prompting generally provides stronger performance than the corresponding zero-shot setting across the evaluated dataset–backbone combinations, indicating that additional in-context demonstrations can improve answer discrimination without modifying model parameters. Compared with standard LoRA, introducing either OLKS or RADA consistently improves the reported evaluation metrics, showing that both candidate-specific structured knowledge modeling and relation-aware dynamic adaptation make useful individual contributions to the framework. Their relative effects vary across the two backbone models: RADA generally shows a stronger contribution with Llama3 8B, whereas OLKS is comparatively stronger with Qwen 7B. This difference suggests that the two components capture complementary aspects of the medical question-answering task rather than providing identical forms of improvement. When OLKS and RADA are jointly incorporated, the complete framework achieves the strongest overall performance across all evaluated dataset–backbone settings and metrics, supporting the complementary role of structured option-level knowledge representation and candidate-conditioned dynamic adaptation in the proposed framework.

### 4.6 Visualize experimental results

#### 4.6.1 Case-level visualization of option-level knowledge subgraphs.

To provide an intuitive illustration of the knowledge modeling process of the proposed method in medical multiple-choice question answering, representative cases are selected from the test samples, and the option-level knowledge subgraphs corresponding to different candidate answers are visualized. For each candidate, the visualization presents the local structural relationships among question-related entities, the candidate-specific option entity, supporting medical concepts, and weak or less relevant concepts. By comparing the connectivity patterns and medical evidence coverage of the correct option with those of the distractor options, the role of option-level knowledge subgraph modeling in candidate discrimination can be more clearly illustrated, as shown in Fig 4.

**Fig 4:**
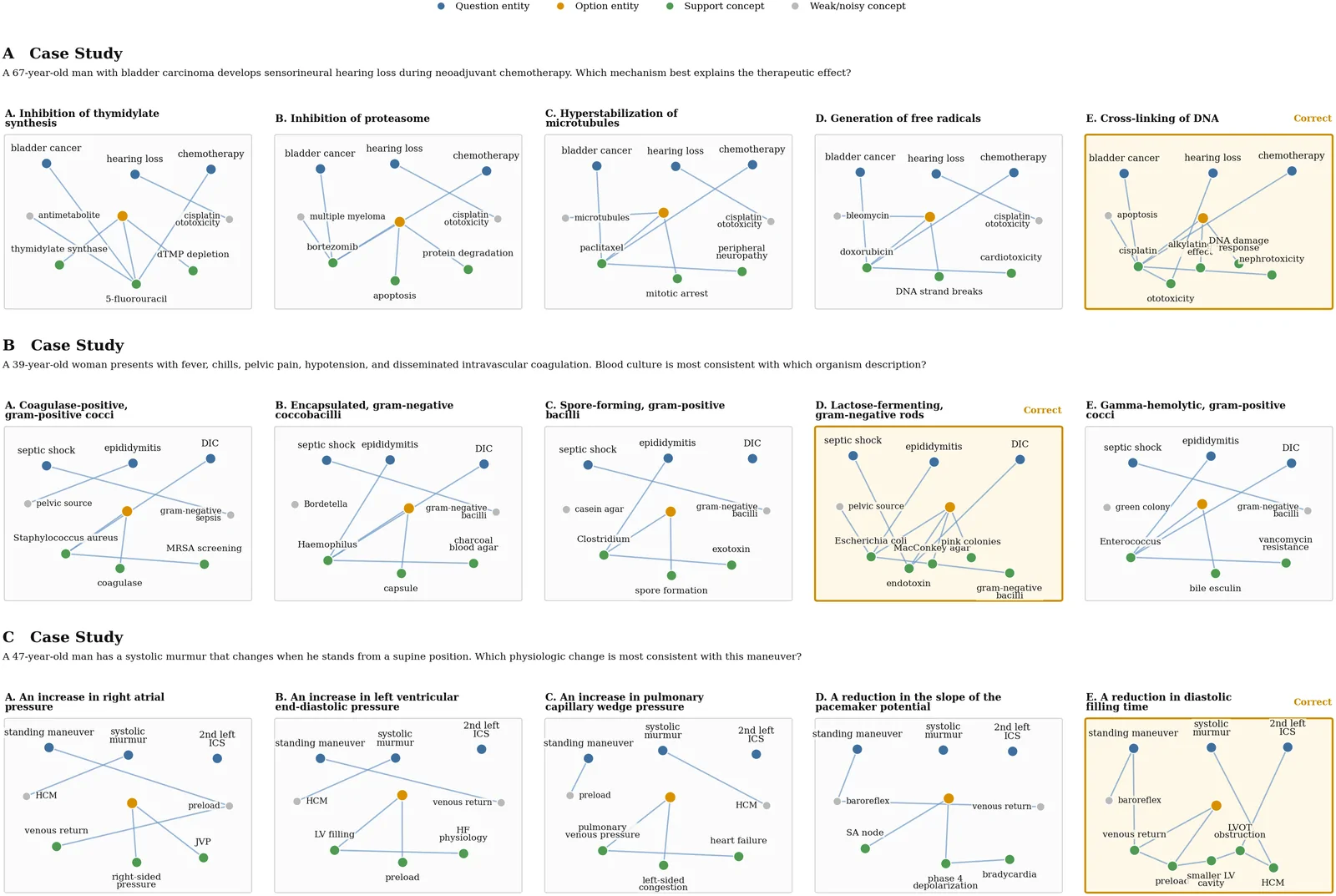
Case-level visualization of option-level knowledge subgraphs, showing candidate-specific structural connections among question entities, option entities, supporting concepts, and weak or less relevant concepts in three representative medical question-answering cases.

As shown in Fig 4, the option-level knowledge subgraphs exhibit distinct candidate-specific structural patterns across the three representative cases. In the selected examples, the correct options are associated with more coherent connections between question-related entities, candidate-specific option entities, and clinically relevant supporting concepts. In contrast, the distractor options may still contain medically related concepts, but their local subgraphs tend to contain fewer directly relevant connections or more weakly related concepts with respect to the clinical information provided in the question stem. These visual differences illustrate how the OLKS module constructs a separate structured representation for each candidate rather than relying solely on textual similarity between the question and the answer options.

More specifically, the correct-option subgraphs in the selected cases contain clinically meaningful paths that connect the candidate option with multiple supporting concepts and question-related entities. Such candidate-specific structural organization provides the subsequent prediction module with additional relational information that complements the semantic representation produced by the language model. The visualization therefore provides qualitative evidence that option-level subgraph construction can preserve differences in medical relational structure across candidate answers and can support more fine-grained candidate discrimination in the proposed framework.

### 4.7 Visualization of option-level knowledge support distribution

To further examine the candidate-specific structural evidence provided by the option-level knowledge subgraphs, we analyze the graph-only knowledge support scores on both MedQA and MedMCQA. The score is computed exclusively from the fixed topology of the candidate-specific UMLS subgraph and does not involve prediction logits, learned graph representations, RADA routing weights, or other quantities optimized by the question-answering objective. Therefore, the complete-set graph-only support distribution is independent of the language-model backbone, whereas the error-specific analysis is performed separately for the Llama3 8B and Qwen 7B backbones because the incorrectly predicted instances depend on the corresponding model predictions.

For the *j*-th question, let $E_{q}^{\left(j\right)}$ and $E_{i}^{\left(j\right)}$ denote the retained UMLS concepts linked from the question stem and the *i*-th candidate option, respectively, and let $G_{j,i}$ denote the corresponding option-level subgraph. The graph-only knowledge support score for candidate *i* is defined as


$$\begin{array}{c} S_{j,i}=\frac{1}{\left|E_{i}^{\left(j\right)}\right|}\sum_{v\in E_{i}^{\left(j\right)}}\max_{u\in E_{q}^{\left(j\right)}}\kappa \;\left(d_{G_{j,i}}(u,v)\right), \end{array}$$

where


$$\begin{array}{c} \kappa (d)=\left\{ \begin{array}{cc} \frac{1}{1+d}, & d\leq 2,\\ {}[4pt]0, & d>2\text{ or no path exists}. \end{array}\right. \end{array}$$

Accordingly, shorter graph paths between question-linked and candidate-linked concepts correspond to stronger structural support. We first compare the gold option with the mean support of all distractors over the complete evaluation set for each dataset. For prediction errors, we further compare the support assigned to the gold option, the incorrectly selected option, and the mean support of the remaining distractors separately for each backbone. Here, the reported number of incorrect predictions denotes pooled prediction instances across the three independent runs rather than the number of unique questions.

As shown in Fig 5, over the complete evaluation sets, the gold options receive higher average graph-based support than the mean distractor support on both datasets. Specifically, the mean graph-only support scores of the gold options and distractors are 0.690 and 0.452 on MedQA, respectively, and 0.625 and 0.508 on MedMCQA, respectively. Because these complete-set scores are determined solely by the fixed UMLS subgraph topology, they are independent of the language-model backbone.

**Fig 5:**
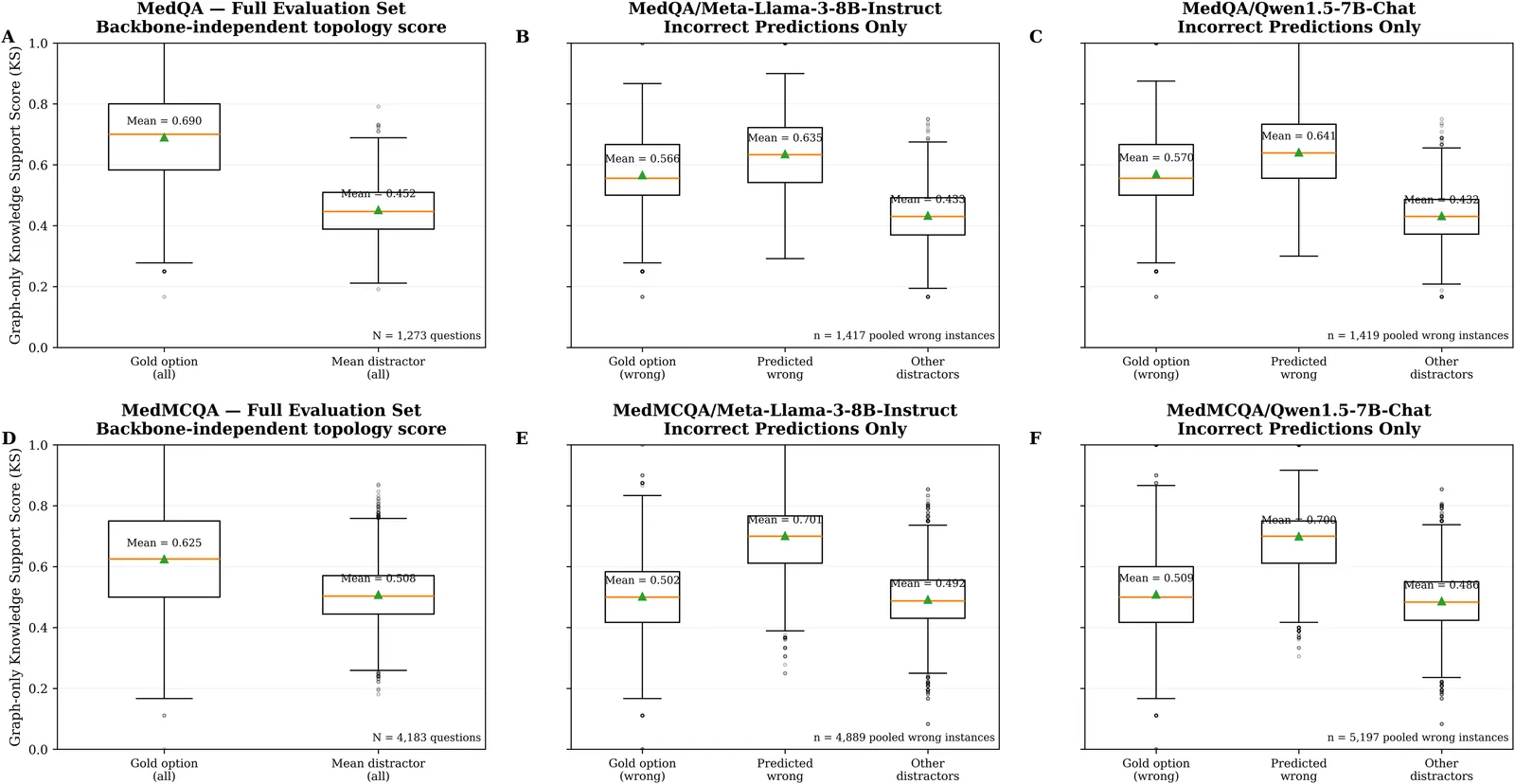
Distribution of graph-only knowledge support scores on MedQA and MedMCQA. Panels A and D show the backbone-independent distributions over the complete evaluation sets, while Panels B, C, E, and F show backbone-specific analyses restricted to incorrectly predicted instances pooled across the three independent runs.

A different pattern emerges when only incorrect prediction instances are considered. In these error-specific subsets, the option selected incorrectly by the model consistently exhibits the highest mean graph-only support, exceeding both the corresponding gold option and the remaining distractors. On MedQA, under Llama3 8B, the mean support scores of the gold option, incorrectly predicted option, and remaining distractors are 0.566, 0.635, and 0.433, respectively; under Qwen 7B, the corresponding values are 0.570, 0.641, and 0.432. On MedMCQA, the corresponding values are 0.502, 0.701, and 0.492 under Llama3 8B, and 0.509, 0.700, and 0.480 under Qwen 7B. These results suggest that the option-level UMLS subgraphs generally provide stronger structural support for the correct answer over the complete evaluation sets, while prediction failures are frequently associated with distractors that also receive comparatively strong graph-based support. This observation reflects ambiguity in the underlying structured medical evidence rather than a deterministic correspondence between graph support and the final model prediction.

### 4.8 Hyperparameter sensitivity experimental results

#### 4.8.1 Sensitivity analysis of Top-K LoRA expert selection.

To further analyze the influence of key hyperparameters in the relation-aware dynamic adaptation module on model performance, this study conducts a sensitivity experiment on the number of activated LoRA expert branches, namely Top-K. Top-K controls the number of learned low-rank expert branches that can be invoked for each sample during dynamic LoRA adaptation, and therefore directly affects the model’s ability to represent heterogeneous medical relation patterns and suppress noise from weakly relevant branches. By conducting comparisons on the MedQA and MedMCQA datasets with Llama3 8B and Qwen 7B as backbone models, the performance variation under different Top-K settings can be comprehensively evaluated, as shown in Fig 6.

**Fig 6:**
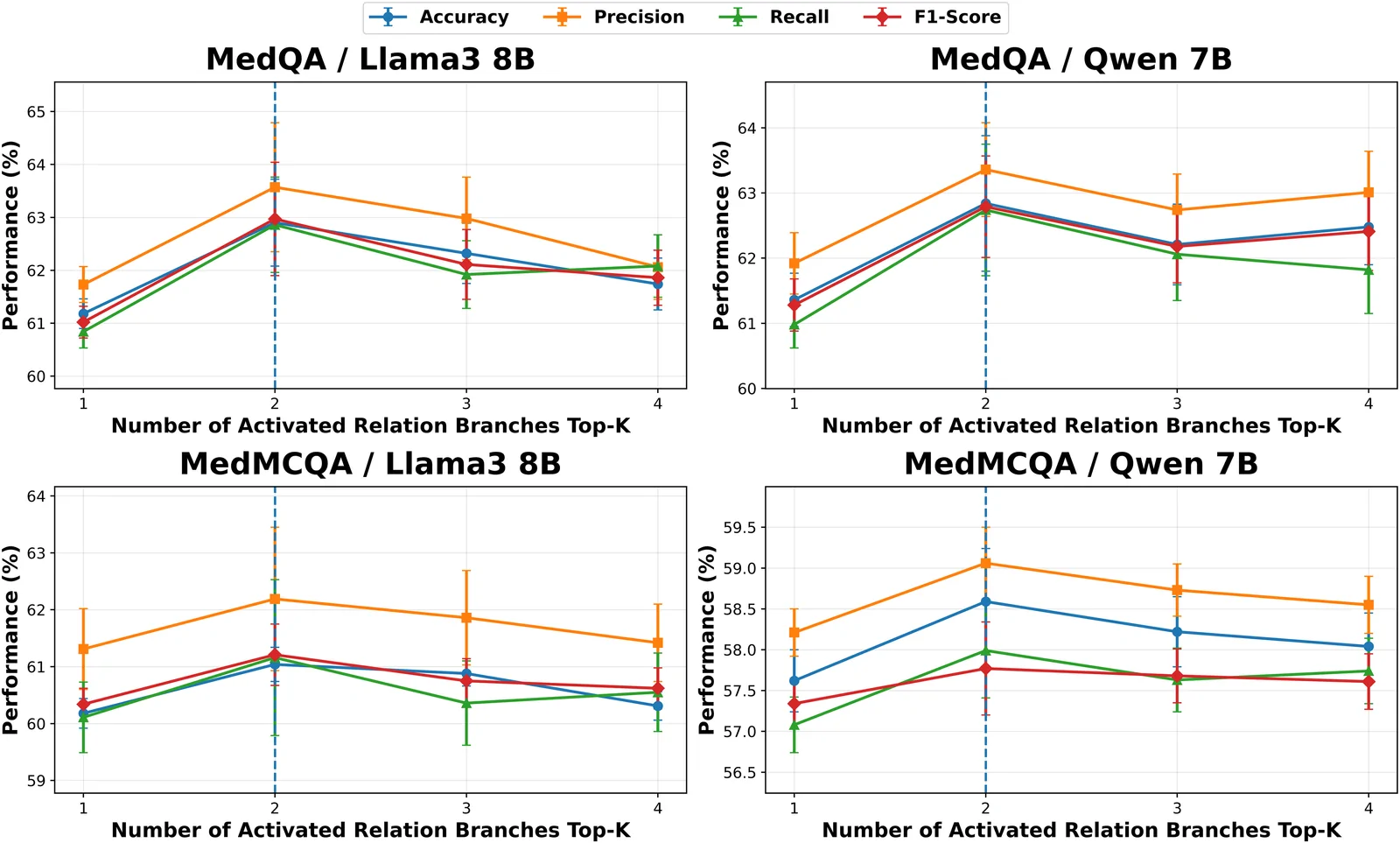
Hyperparameter sensitivity results of Top-K LoRA expert selection on MedQA and MedMCQA with Llama3 8B and Qwen 7B backbones. Results are reported as the mean with standard deviation over three runs.

As shown in Fig 6, model performance varies moderately across the evaluated Top-K settings. The configuration *K* = 2 yields consistently competitive results across the different dataset–backbone combinations, with relatively strong performance across Accuracy, Precision, Recall, and F1-Score. When *K* = 1, each sample is restricted to a single LoRA expert branch during dynamic adaptation, which reduces the diversity of available learned update paths. Increasing *K* allows multiple expert branches to contribute simultaneously, although larger values do not produce consistent performance gains across all settings. Overall, the relatively limited variations and overlapping standard deviations across neighboring configurations indicate that the proposed relation-aware adaptation mechanism maintains stable performance over the evaluated Top-K range.

#### 4.8.2 Sensitivity analysis of LoRA rank.

The LoRA rank parameter *r* determines the representation capacity of the low-rank adaptation matrices and is an important hyperparameter affecting parameter-efficient fine-tuning performance and model stability. For medical multiple-choice question answering tasks, an excessively small rank may limit the model’s ability to capture complex medical semantics and relation patterns, whereas an excessively large rank may introduce redundant updates and weaken the parameter efficiency of low-rank adaptation. Therefore, this study further investigates the performance variation of the model under different LoRA rank settings across different datasets and backbone models, as shown in Fig 7.

**Fig 7:**
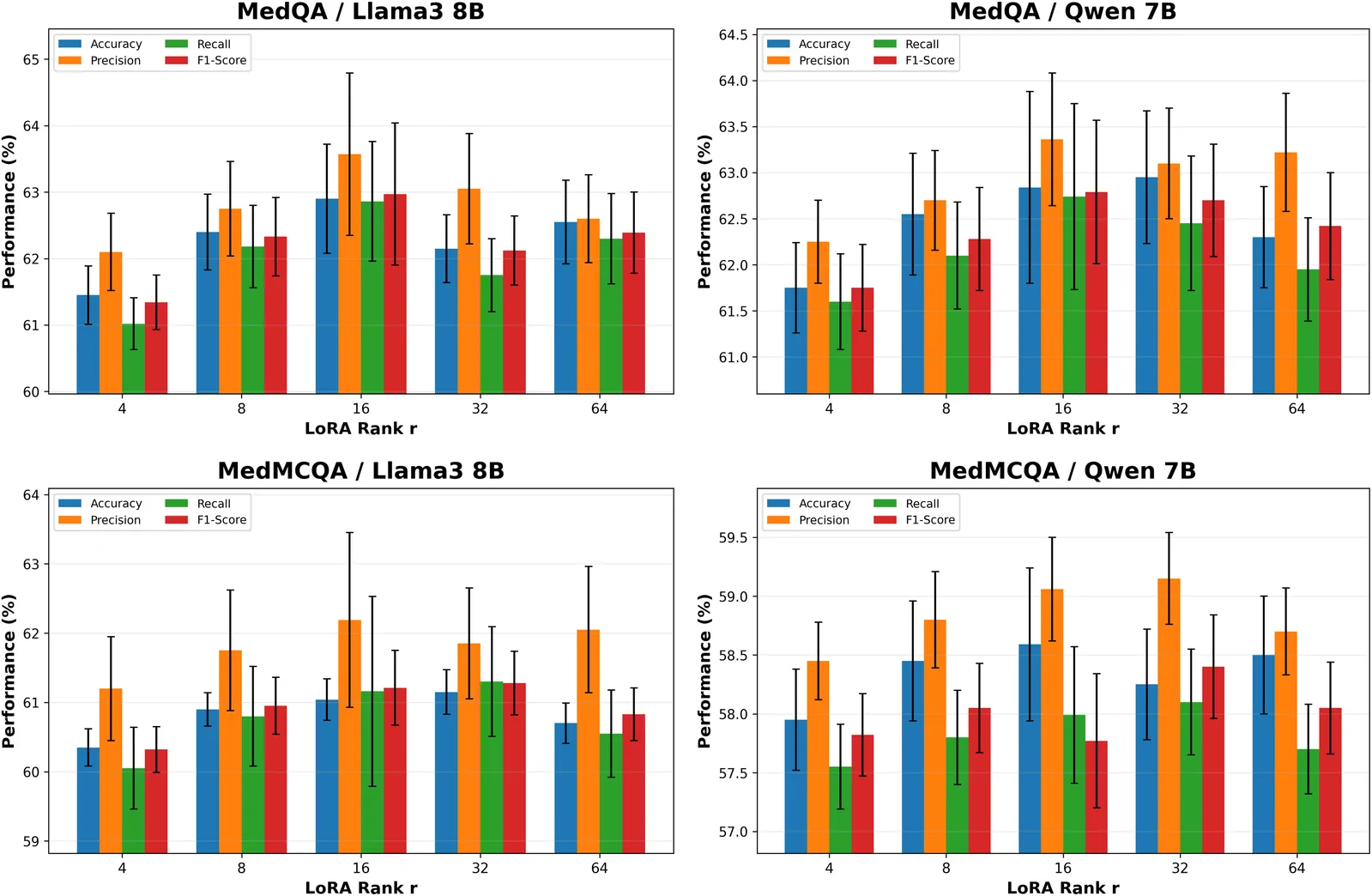
Hyperparameter sensitivity results of LoRA rank on MedQA and MedMCQA with Llama3 8B and Qwen 7B backbones. Results are reported as the mean with standard deviation over three runs.

As shown in Fig 7, the model exhibits moderate performance variation across the evaluated LoRA rank settings. The configuration *r* = 16 achieves consistently competitive performance across the two datasets and two backbone models, while several neighboring rank settings also produce comparable results. Lower ranks provide a more constrained low-rank update space, whereas increasing the rank enlarges the adaptation capacity but does not lead to consistent improvements across all settings. In particular, the differences among several neighboring rank configurations remain relatively small relative to their standard deviations. These results indicate that the proposed framework is not highly sensitive to moderate changes in the LoRA rank within the evaluated range.

## 5 Discussion and limitations

This study focuses on the challenges of semantically similar candidate answers, complex medical relations, and the difficulty of unified parameter adaptation in capturing heterogeneous reasoning patterns in medical multiple-choice question answering, and proposes a knowledge-enhanced large language model framework that integrates option-level knowledge subgraph representation fusion with relation-aware dynamic LoRA adaptation. The experimental results show that option-level knowledge subgraphs can construct independent structured medical evidence for different candidate answers, enabling the model to form a more fine-grained relational discrimination basis between question-stem entities and candidate entities; meanwhile, the relation-aware dynamic adaptation mechanism further maps such relational differences to the parameter update level, allowing different candidate representations to activate differentiated combinations of learned low-rank adaptation paths. Compared with existing parameter-efficient fine-tuning methods, the proposed method not only improves overall prediction performance, but also enhances the interpretability and stability of the candidate answer discrimination process. To contextualize these results with published models of a comparable parameter scale, recent studies have reported substantial variation in MedQA performance across 7–8B models under different training and inference protocols. For example, KGARevion reported an accuracy of 56.6% for LLaMA3-8B on MedQA-US under its multiple-choice reasoning setting [37], whereas Meerkat-7B achieved 71.2% in single-model evaluation and 77.1% with ensemble inference after incorporating medical chain-of-thought supervision and textbook-derived training data [38]. These results indicate that absolute performance across studies can vary substantially with backbone configuration, reasoning supervision, additional medical training data, and inference strategy. Therefore, cross-study numerical comparisons should be interpreted cautiously, while controlled comparisons under consistent backbone models and evaluation protocols provide a more direct basis for assessing the proposed framework. However, there remains room for further improvement. On the one hand, the model performance depends to some extent on the coverage of external medical knowledge graphs and the quality of entity linking; when rare medical concepts appear in the question stem or candidate options, the local knowledge subgraphs may not fully cover all potential evidence. On the other hand, the current experiments mainly focus on medical multiple-choice question answering scenarios. Although the effectiveness of the proposed method in structured medical reasoning has been verified, its applicability to open-ended medical question answering, multi-turn clinical question answering, and more complex medical reasoning tasks still requires further exploration. Future research can introduce higher-quality medical knowledge resources and more flexible knowledge update mechanisms while maintaining parameter efficiency, thereby further improving the generalization ability and practical value of the model in real-world medical question answering scenarios.

## 6 Conclusion

This study proposes a knowledge-enhanced large language model framework for medical multiple-choice question answering, which improves the model’s ability to identify fine-grained medical relation differences among candidate answers through the collaborative modeling of option-level knowledge subgraph representation fusion and relation-aware dynamic LoRA adaptation. The framework first constructs a local medical knowledge subgraph for the question stem and each candidate option, and introduces structured knowledge into candidate answer representations through graph neural network encoding and a gated fusion mechanism, thereby enhancing the model’s ability to discriminate medical entity associations, evidence paths, and candidate plausibility. On this basis, the relation-aware dynamic LoRA adaptation mechanism adaptively combines learned low-rank expert branches according to the candidate-conditioned relational representations, enabling different candidate options to obtain differentiated parameter update paths. Experimental results show that the proposed method outperforms multiple comparison methods on the MedQA and MedMCQA datasets, while ablation experiments and visualization analyses further verify the effectiveness of option-level knowledge modeling and dynamic relation adaptation in improving medical question answering performance and enhancing reasoning interpretability.

Future research can further explore more fine-grained and continuously updated medical knowledge enhancement mechanisms, enabling the model to more fully utilize dynamic knowledge information from clinical guidelines, drug knowledge bases, and medical literature. Meanwhile, the proposed framework can also be extended to more complex tasks, such as open-ended medical question answering, medical diagnosis and treatment recommendation generation, clinical decision support, and multimodal medical reasoning, so as to further examine the generalization ability of option-level knowledge modeling and relation-aware parameter adaptation across different medical intelligence application scenarios. With the continuous improvement of medical knowledge graph construction quality, entity linking techniques, and large language model reasoning capabilities, the deep integration of structured medical knowledge and parameter-efficient adaptation mechanisms is expected to provide more stable technical support for reliable, interpretable, and efficient medical question answering systems.

## Supporting Information

S1 File Appendix. Supplementary paired bootstrap significance analysis and cost experimental results.
(DOCX)
