# Supplementary material for: Relation-aware dynamic knowledge subgraph enhanced large language models for medical multiple-choice question answering
Source: PLoS One. 2026 Oct 16;21(10):e0349628. doi: 10.1371/journal.pone.0349628 (PMC13674765; doi:10.1371/journal.pone.0349628)
Supplement: Supplementary file 1 [file pone.0349628.s001.docx]

| **Dataset** | **Backbone** | **Baseline** | **ΔAcc (%)** | **95% CI** | **Holm-adjusted p** |
| --- | --- | --- | --- | --- | --- |
| MedQA | Meta-Llama-3-8B-Instruct | Full Fine-Tuning | 1.31 | [-0.68, 3.30] | 0.594 |
| MedQA | Meta-Llama-3-8B-Instruct | Towards Specialized Generalists | 1.26 | [-0.68, 3.27] | 0.594 |
| MedQA | Meta-Llama-3-8B-Instruct | AMG-RAG | 1.49 | [-0.52, 3.59] | 0.594 |
| MedQA | Meta-Llama-3-8B-Instruct | HydraLoRA | 1.31 | [-0.65, 3.38] | 0.594 |
| MedQA | Qwen1.5-7B-Chat | Full Fine-Tuning | 1.20 | [-0.76, 3.19] | 0.420 |
| MedQA | Qwen1.5-7B-Chat | Towards Specialized Generalists | 1.26 | [-0.73, 3.25] | 0.420 |
| MedQA | Qwen1.5-7B-Chat | AMG-RAG | 1.75 | [-0.24, 3.74] | 0.254 |
| MedQA | Qwen1.5-7B-Chat | HydraLoRA | **3.72** | **[1.70, 5.73]** | **0.002** |
| MedMCQA | Meta-Llama-3-8B-Instruct | Full Fine-Tuning | 1.23 | [-0.28, 2.73] | 0.110 |
| MedMCQA | Meta-Llama-3-8B-Instruct | Towards Specialized Generalists | 1.34 | [0.02, 2.65] | 0.094 |
| MedMCQA | Meta-Llama-3-8B-Instruct | AMG-RAG | **1.57** | **[0.40, 2.73]** | **0.022** |
| MedMCQA | Meta-Llama-3-8B-Instruct | HydraLoRA | **2.82** | **[1.71, 3.94]** | **<0.001** |
| MedMCQA | Qwen1.5-7B-Chat | Full Fine-Tuning | **1.31** | **[0.14, 2.49]** | **0.048** |
| MedMCQA | Qwen1.5-7B-Chat | Towards Specialized Generalists | **1.85** | **[0.48, 3.22]** | **0.023** |
| MedMCQA | Qwen1.5-7B-Chat | AMG-RAG | **1.33** | **[0.20, 2.45]** | **0.048** |
| MedMCQA | Qwen1.5-7B-Chat | HydraLoRA | **2.38** | **[0.92, 3.78]** | **0.006** |
